# The BNT162b2 vaccine induces humoral and cellular immune memory to SARS-CoV-2 Wuhan strain and the Omicron variant in children 5 to 11 years of age

**DOI:** 10.3389/fimmu.2022.1094727

**Published:** 2022-12-15

**Authors:** Bianca Laura Cinicola, E Piano Mortari, Anna Maria Zicari, Chiara Agrati, Veronica Bordoni, Christian Albano, Giorgio Fedele, Ilaria Schiavoni, Pasqualina Leone, Stefano Fiore, Martina Capponi, Maria Giulia Conti, Laura Petrarca, Paola Stefanelli, Alberto Spalice, Fabio Midulla, Anna Teresa Palamara, Isabella Quinti, Franco Locatelli, Rita Carsetti

**Affiliations:** ^1^ Department of Maternal Infantile and Urological Sciences, Sapienza University of Rome, Rome, Italy; ^2^ Department of Molecular Medicine, Sapienza University of Rome, Rome, Italy; ^3^ B cell unit, Immunology Research Area, IRCCS Bambino Gesù Children’s Hospital, Rome, Italy; ^4^ Department of Pediatric Hematology and Oncology and Cell and Gene Therapy, IRCCS Bambino Gesù Children’s Hospital, Rome, Italy; ^5^ Department of Infectious Diseases, Istituto Superiore di Sanità, Rome, Italy; ^6^ Catholic University of the Sacred Hearth, Rome, Italy

**Keywords:** SARS-CoV-2, Omicron, vaccine, children, immune memory, memory B cells, antigen-specific T cells, antibodies

## Abstract

SARS-CoV-2 mRNA vaccines prevent severe COVID-19 by generating immune memory, comprising specific antibodies and memory B and T cells. Although children are at low risk of severe COVID-19, the spreading of highly transmissible variants has led to increasing in COVID-19 cases and hospitalizations also in the youngest, but vaccine coverage remains low. Immunogenicity to mRNA vaccines has not been extensively studied in children 5 to 11 years old. In particular, cellular immunity to the wild-type strain (Wuhan) and the cross-reactive response to the Omicron variant of concern has not been investigated. We assessed the humoral and cellular immune response to the SARS-CoV-2 BNT162b2 vaccine in 27 healthy children. We demonstrated that vaccination induced a potent humoral and cellular immune response in all vaccinees. By using spike-specific memory B cells as a measurable imprint of a previous infection, we found that 50% of the children had signs of a past, undiagnosed infection before vaccination. Children with pre-existent immune memory generated significantly increased levels of specific antibodies, and memory T and B cells, directed against not only the wild type virus but also the omicron variant.

## Introduction

SARS-CoV-2 infection presents with a wide spectrum of clinical manifestations, ranging from mild to severe systemic disease. Most children are either asymptomatic or develop mild manifestations, with rare pulmonary involvement (1). Recently, however, after the appearance of the high transmissibility of the Omicron Variant of Concern (VOC), the number of infections and hospitalizations has also increased in the 5 to 11 years old age group (2). According to the Italian National Institute of Health (ISS) reports, in the last week of August 2022, COVID-19 infections in individuals below 18 years of age accounted for 12% of the recorded cases (3).

Although most of the infected children and adolescents are asymptomatic, severe and sometimes life-threatening complications may occur. In particular, the development of the Multisystem Inflammatory Syndrome in Children (MIS-C), a severe post-infectious hyperinflammatory condition, has been reported to present 2-6 weeks after a typically mild or asymptomatic infection (4). Moreover, the persistence of symptoms long after infection, the so-called “long-COVID”, should not be underestimated (5). Pediatric COVID-19 also poses public health concerns as transmission from children may cause severe disease in adult household members (6). Furthermore, the COVID-19 pandemic negatively impacts the psychosocial well-being of children and their families, particularly those with special education needs and lower socio-economic status (7).

Vaccines are the only effective large-scale tool to prevent infection and control the pandemic. Over the last two years, several vaccines against SARS-CoV-2 have been rapidly developed. To date, all commercially available vaccines have been shown to be protective against hospitalization and serious illness after completing the immunization schedule (8). In the pediatric population, only mRNA vaccines have been approved, first for adolescents aged 12 to 15 years for Pfizer BNT162b2 (9) and 12 to 17 years for Moderna mRNA-1273 vaccine (10) and later for children between 5 and 11 years of age (11, 12). Vaccine use in children 6 months through 4 years of age has also been approved by the FDA (13). In Italy, mRNA vaccines have been introduced in late December 2021 for children aged 5 to 11.

The administration of two doses of the Pfizer-BioNTech vaccine in children aged 5-11 years has been proven to be safe, effective, and capable of eliciting a robust humoral immune response to SARS-CoV-2, comparable to that of the adolescents (11).

Specific B- and T-cell memory induced by mRNA vaccines has not been investigated in this population.

Vaccines protect from infection because they induce immunological memory (14). Whereas serum antibodies significantly decline a few months after the last SARS-CoV-2 vaccine dose (15, 16), memory B (17) and T (18) cells persist and increase. In case of infection, pre-formed serum antibodies exert the first-line immediate protection, meanwhile memory B cells rapidly expand and differentiate into plasmablasts responsible for the rapid increase of specific antibodies in the serum (14). Memory cells also migrate to the site of viral entry (19). Here, memory B cells secrete neutralizing antibodies that prevent viral spreading, and T cells kill virus-infected cells (17, 20, 21).

The failure of COVID-19 vaccines to prevent infection and contagion has been attributed to their inability of inducing mucosal immune protection (17, 22, 23) and to the emergence of variants of concern (VOC) (24) with increased transmissibility.

However, the third dose of COVID-19 vaccines strongly boosts immunity and, at the population level, reduces severe COVID-19-associated morbidity and mortality, thanks to the increase of antibodies and memory B and T cells able to neutralize not only the original Wuhan strain but also the other variants that appeared over time (15, 25–27).

In a recent study conducted on children and adolescents, vaccination reduced the risk of hospitalization for COVID-19 by two-thirds in children 5 to 11 years old during the Omicron period, while most children with critical courses were unvaccinated (28). In adolescents, although the effectiveness of two doses of the BNT162b2 vaccine against hospitalization was lower during the Omicron than the Delta wave, vaccination prevented most life-threatening COVID-19 cases in both periods (28). Vaccine effectiveness among adolescents increased after a booster dose (29).

Recently, different rates of effectiveness of two doses of the BNT162b2 vaccine have been reported in children from Italy and Singapore. The effectiveness against hospitalization was 41.1% in Italy (30) and 82.7% in Singapore (31) during the Omicron period. The discrepancy between these results may be due to different criteria for hospitalization in the two countries.

In this study, we describe the humoral and cell-mediated immune response in children, in order to comprehensively evaluate vaccine immunogenicity in this population. A broad understanding of how SARS-CoV-2 vaccination activates the immune system is necessary to find the best predictors of long-term protection and identify individuals that would benefit from additional vaccine doses also in the pediatric age.

Moreover, our understanding of the immunological features associated with the main VOCs will be helpful to inform health policies, including boosting and vaccination schedules.

## Methods

### Study design and patients

We conducted a cross-sectional study on 30 healthy subjects aged 5 to 11 years old enrolled from February to March 2022 at the pediatric Vaccination Center of Policlinico Umberto I (Sapienza University of Rome) where they received Pfizer-BioNTech (BNT162b2) mRNA vaccine immunization.

We excluded subjects who already received one or two doses of Pfizer-BioNTech COVID-19 vaccine prior to enrollment, subjects diagnosed with primary or secondary immunodeficiency or with an ongoing infection and, children taking any immunosuppressive drug.

The SARS-CoV-2 vaccine was administered as prescribed, in two doses of 10 μg, 21 days apart. Two blood samples were obtained from each participant for serological and cellular immunity assessment at time 0 (T0), before the first dose, and 7-15 days after the second dose (T1).

The study protocol was approved by the Ethical Review Committee of Sapienza, University of Rome, Italy (Prot. 0254/2022). The study was performed in accordance with the Good Clinical Practice guidelines, the International Conference on Harmonization guidelines, and the most recent version of the Declaration of Helsinki.

Parents of the eligible patients were informed on the study, including its safety profile and supply procedures, and signed the informed consents for vaccination and for the immunological study. A structured questionnaire was administered to the parents to investigate whether the children had had positive NPS or experienced COVID-19 before.

Demographic (age, gender, and ethnicity) and clinical data were collected to assess the conditions of these subjects (**Table 1**).

**Table 1:** Demographic and immunological characteristics of the enrolled children.

		Enrolled children	Group 1	Group 2	p value [Group 1 vs Group 2]
	N	27	15	12	
	Age (mean, SD)	8.1 (2.3)	7.8 (2.3)	8.5 (2.3)	
	Gender	17F/10M	8F/7M	9F/3M	
	Ethnicity	11 Caucasian; 12 Asian; 3 American; 1 African	6 Caucasian; 7 Asian; 1American; 1 African	5 Caucasian; 5 Asian; 2 American	
	Positive NPS (pre-vaccine)	3 [11.1%]; mild symptoms	3 [11.1%]; mild symptoms	0%	
Anti-N IgG	T0	0.86 [0.24-3.7]	1.99 [0.75-4.8]	0.24 [0.1-0.8]	0.002
	T1	0.45 [0.22-1.3]	0.3 [0.11-1.13]	0.22 [0.1-0.7]	0.02
Anti-TrimericS IgG BAU/ml	T0	63.1 [6-929]	255 [163-1430]	5.430 [4.8-31.3]	<0.0001
	T1	8380 [5120-11800]	11200 [8120-26400]	5765 [2405-8388]	0.001
Neutralization Wuhan IC50	T0	16 [8-160]	160 [64-256]	8 [8-8]	<0.0001
	T1	1024 [1024-1024]	1024 [1024-1024]	1024 [640-1024]	0.047
Inhibitory activity Omicron	T0	0.1 [0.1-10]	10 [0.1-10]	0.1 [0.1-0.1]	0.0065
	T1	30 [10-90]	30 [30-90]	10 [0.1-03]	0.01
Spike-specific memory B cells [%]	T0	0.017 [0-0.08]	0.06 [0.03-0.12]	0 [0-0]	<0.0001
	T1	0.1 [0.04-0.3]	0.23 [0.09-0.84]	0.038 [0.01-0.1]	0.0002
T cells specific for Wuhan Spike [SFC/10^6]	T0	133 [11.5-338]	137 [36-330.5]	47 [0-677]	ns
	T1	563 [154-1985]	588 [358-2409]	174 [133-1207]	ns
T cells specific for Omicron Spike [SFC/10^6]	T0	7 [0-43.5]	20 [0-68.5]	0 [0-17]	ns
	T1	27 [5-140]	91.5 [17.2-264.5]	17 [3-27]	0.0479
T cells specific for Wuhan reference Spike [SFC/10^6]	T0	0 [0-36.5]	6.5 [0-83.2]	0 [0-3]	ns
	T1	40 [3.5-150]	131.5 [31.7-351]	20 [0-40]	0.0108

ns means not significant.

### Cell isolation and cryopreservation

Peripheral blood mononuclear cells (PBMCs) were isolated by Ficoll Paque™ Plus 206 (Amersham PharmaciaBiotech, Amersham, UK) density-gradient centrifugation and immediately frozen and stored in liquid nitrogen until use.

### Detection of SARS-CoV-2 specific antibodies

SARS-CoV-2 specific antibodies were detected on plasma with the DiaSorin Liaison SARS-CoV-2 TrimericS IgG assay (DiaSorin, Saluggia, Italy). The assay was performed on the LIAISON^®^ XL chemiluminescence analyzer.

Anti-Nucleocapsid IgG were measured by Anti-SARS-CoV-2 NCP ELISA assay (Euroimmun, Lübeck, Germany), which uses a modified nucleocapsid protein that only contains diagnostically relevant epitopes. The assay was performed on an automated I-2P analyzer (Euroimmun, Lübeck, Germany).

### Neutralizing antibodies against the Wuhan SARS-CoV-2 strain

The Wuhan SARS-CoV-2 strain [GF1] (B1) was incubated at 100 TCID50 (Median Tissue Culture Infectious Dose) with two-fold serial dilutions of plasma samples (1:8 to 1:512) to determine the microneutralization titer (MNT). All plasma samples were first heat-treated for 30 minutes at 56°C to inactivate complement. Virus-plasma mixtures were kept at 37°C for 1 hour in EMEM cell culture medium (Sigma Aldrich, St. Louis, USA) supplemented with 1X penicillin/streptomycin (Corning, Glendale, USA) and 2% fetal bovine serum (Corning, Glendale, USA) in 96-well plates. After the incubation, 22.000 Vero E6 cells (ATCC^®^ CRL-1586) were added to each well and cultured at 37°C for 5 days. MNT was calculated as the serum dilution capable of reducing the cytopathic effect to 50%. Positive and negative serum samples and cell culture control were included in each test.

### Antibody inhibitory activity of the binding between B.1.1.529 BA.1 RBD and ACE-2

Antibodies able to inhibit the binding of ACE-2 to the B.1.1.529 BA.1 RBD were measured using a colorimetric assay kit (BPSBioscience, San Diego, USA). Briefly, 96 well plates were coated with the RBD protein of the B.1.1.529 BA.1 Variant (1ug/ml). The next day, plasma was added in four 1:3 serial dilutions (1:10 to 1:270). After, we added biotinylated ACE-2 and, after washing, streptavidin-HRP. The plasma dilution capable of reducing the absorbance of the positive control by 50% is reported.

### Detection of SARS-CoV-2-specific B cells

Detection of antigen-specific memory B cells was performed as previously published (16, 17). Briefly, recombinant biotinylated SARS-CoV-2 spike protein (S1+S2; aa16-1211) was purchased from R&D systems (BT10549) and mixed with streptavidin BUV395 or streptavidin PE (BD Bioscience) at 25:1 ratio and 20:1 ratio respectively for 1 hour at 4C. Streptavidin PE-Cy7 (BD Bioscience) was used as a decoy probe to gate out streptavidin-binding B cells. Previously frozen PBMC samples were stained with 100ng of spike-PE, 100ng of spike-BUV395 and 2 ng of streptavidin PE-Cy7 at 4C for 30 min. Following a wash step, a combination of fluorescent antibodies: CD19-BUV737, CD24-BV711, CD27-BV510, CD38-BV421 and IgM-APC was used for surface staining. Spike-specific memory B cells were identified as CD19+CD24+CD27+PeCy7-PE+BUV395+ (Double positive spike; **Supplementary Figure 1**). Samples were acquired on FACS Symphony (BD Bioscience) and analyzed using FlowJo10.7.1 (BD Bioscience).

### Detection of SARS-CoV-2-specific T-cell response

The frequency of spike-specific T cells was assessed by standard IFN gamma ELISpot. PBMC were plated at 3×10^5^ cells/well in ELISpot plates (Human IFN-gamma ELISpot plus kit; Mabtech, Nacka Strand, Sweden) and stimulated with a pool of peptides spanning the whole spike protein of the Wuhan SARS-CoV2 strain, or with a pool of peptides spanning the mutated portion of the Omicron spike protein and, as a control, with a pool of peptides spanning the same region of Wuhan strain spike protein (Miltenyi Biotech, Bergisch Gladbach, Germany). Results are expressed as spot-forming cells (SFC)/10^6^ PBMCs. Cut-off value was set calculating the mean of the background + 2 SD.

### Statistical methods

Patients’ characteristics are summarized in **Table 1**. Immunological variables were compared between the different study times. The data were first tested for normality and homoscedasticity using Shapiro Wilk and Levene’s tests and since the assumptions were violated, non-parametric tests were used for the analysis. The Wilcoxon matched pair signed-rank test or the two-tailed Mann–Whitney U-test were used. Categorical variables were compared by Chi-Square exact test. A two-sided p value less than 0.05 was considered to be statistically significant. All statistical analyses were done using GraphPad Prism 9.3.1 (GraphPad Software).

## Results

### Subject characteristics

**Figure 1:**
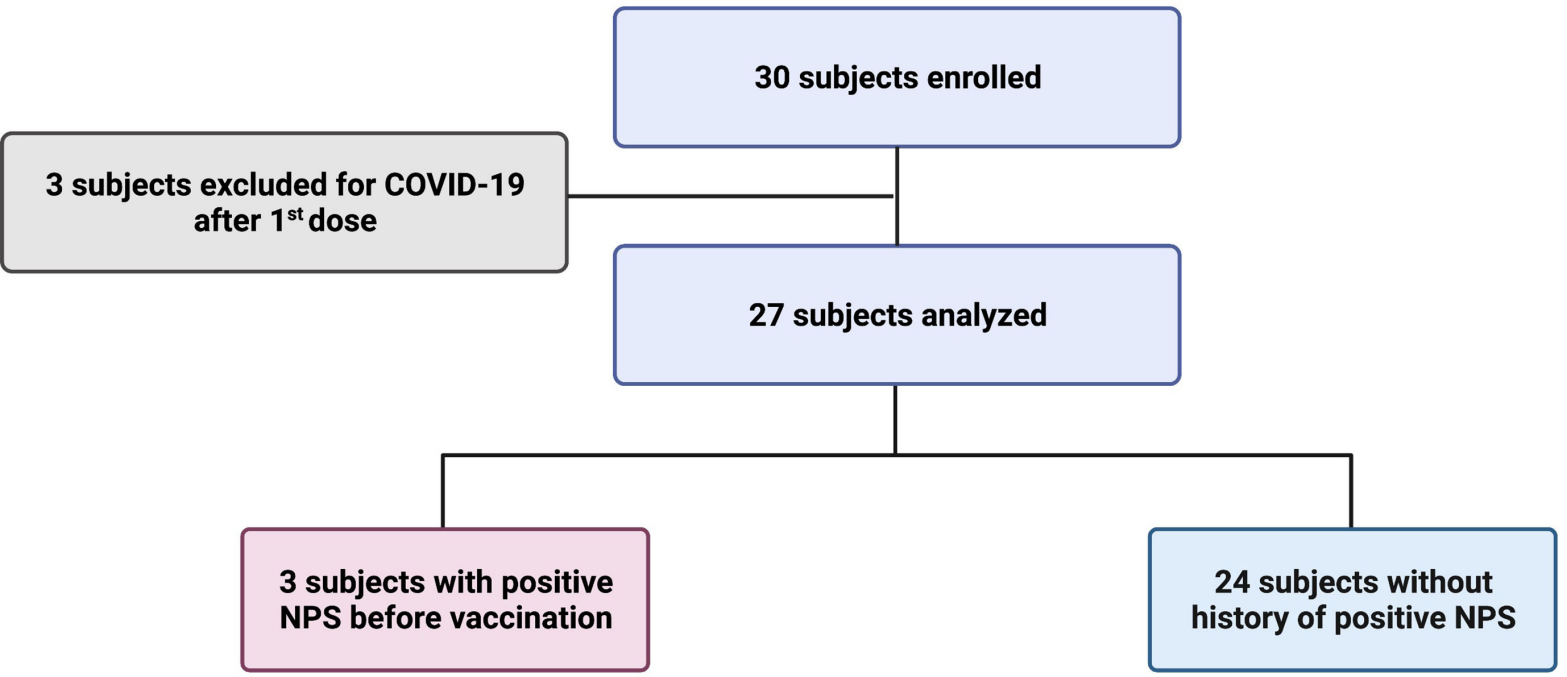
Design of the study.

Of the 30 children enrolled in the study, three were excluded because they had COVID-19 between the first and second dose (**Figure 1**). Of the 27 studied, 11 children were male and 16 were female, with a mean age of 8.1 years (SD 2.3) (**Table 1**). Three subjects (3/27; 11.1%) reported a history of SARS-CoV-2 infection before vaccination (positive molecular Nasopharyngeal Swab, NPS), one 5 months before vaccination and the other two more than a year before. Based on the epidemiology of SARS-CoV-2 in Italy, none of the three children contracted the disease during the Omicron wave. Symptoms were mild (fever, cough, cold, vomit) and resolved in a few days. The infection was detected at the same time in their families. The other 24 children reported no history of infection (**Table 1**).

### Humoral and cellular SARS-CoV-2 response after vaccination

**Figure 2:**
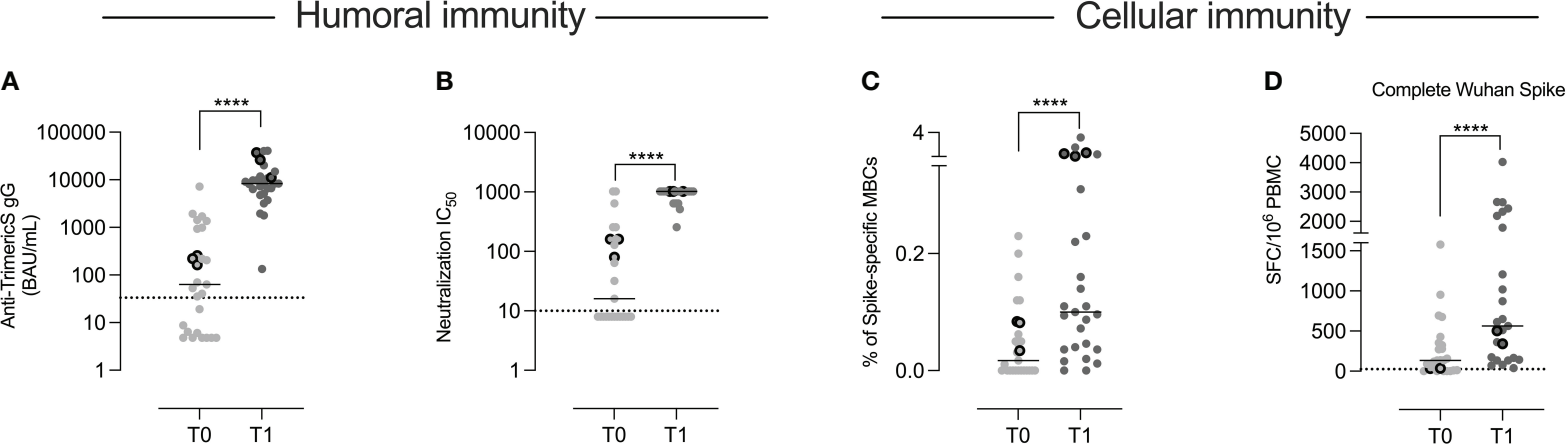
Humoral and cellular immunity. In the 27 children included in the study, we measured the concentration of anti-TrimericS IgG (BAU/mL) before (T0) and 10 days after the 2nd vaccine dose (T1) **(A)**. **(B)** The titer of neutralizing antibodies against the Wuhan virus (IC50) is shown. The frequency of memory B cells specific for the Wuhan spike protein is reported in **(C)**. In **(D)** we show the number of T cells (expressed as numbers of spot-forming cells (SFC)/10^6^ PBMC) that produced IFN gamma against the complete Wuhan spike protein. The three children that had experienced COVID-19 before vaccination are indicated by the dots with thick borders. Dashed lines indicate the cut-off value for each test. Bars indicate medians. A non-parametric Wilcoxon matched pair signed-rank test was used to evaluate statistical significance between T0 and T1. Two-tailed P value significances are shown as ****p < 0.0001.

Anti-TrimericS specific IgG increased significantly after the second dose (T1) compared to T0 (p <0.0001) (**Figure 2** **A**) in all children.

In order to evaluate the quality of the antibody response, we measured the neutralization activity against the Wuhan SARS-CoV-2 strain (**Figure 2** **B**). The increase was significant in all vaccinated children (p<0.001).

By flow-cytometry, we measured the frequency of memory B cells able to bind the Wuhan spike protein (**Supplementary Figure 1**). Specific memory B cells significantly expanded (p <0.0001) (**Figure 2** **C**) after the second dose.

Spike-specific T cells able to produce IFN-gamma after stimulation with peptides spanning the whole Wuhan spike protein (Complete Wuhan spike) were detected by ELISpot. Following vaccination, there was a significant increase of IFN-gamma-secreting T cells (p<0.0001) (**Figure 2** **D**).

As shown in **Figure 2**, at T0, anti-TrimericS were detectable in 16 children and neutralizing antibodies in 12. Spike-specific memory B and T cells were also measurable in some of the samples collected before vaccination. Since, based on their medical history, only three of the 27 children had experienced COVID-19 before (indicated by the dots with thick borders), we asked the question of whether the presence of pre-existing humoral and cellular immunity might be due to previous undiagnosed SARS-CoV-2 infections. Whereas serum antibodies may have been generated in response to other coronaviruses and cross-reactive T cells can be found in individuals never exposed to SARS-CoV-2 (32), spike-specific memory B cells are only detected in individuals who had experienced COVID-19 before or were fully vaccinated (16, 33). Indeed, the presence of specific memory B cells represent a reliable imprint of past contact with a defined antigen because their generation is a complex process occurring in the germinal center (17). In these vaccine-induced structures, immunoglobulin genes are modified by the introduction of somatic mutations, followed by a strict selection for the ability to bind the stimulating antigen. Spike-specific memory B cells persist and continue to increase for months after vaccination (17) or infection (34), ^in^ contrast to specific anti-S and anti-N antibodies that decline over time (35).

Based on these observations, we re-analyzed the data by comparing children who had (group 1) or not (group 2) spike-specific memory B cells at T0 (**Table 1**).

**Figure 3:**
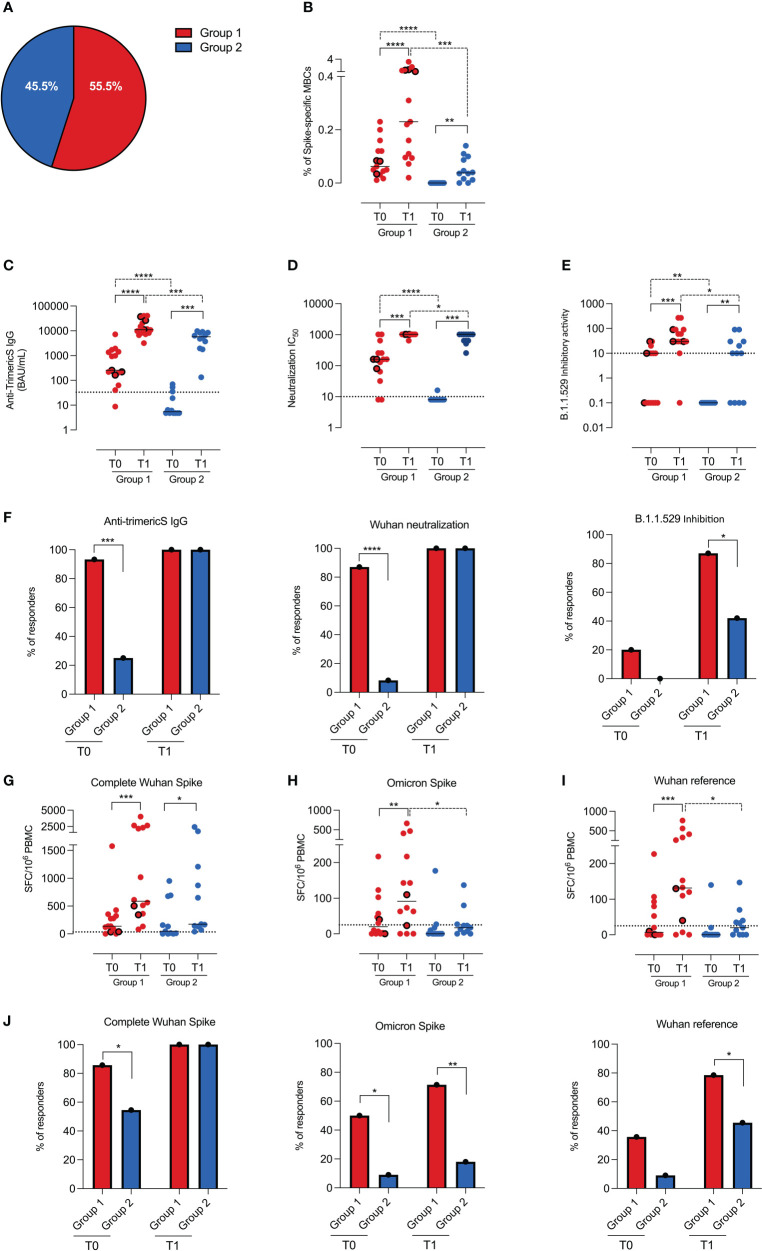
Humoral and cellular immunity in groups 1 and 2. **(A)** Pie chart represents the percentage of children with (group 1, n=15) and without (group 2, n=12) spike-specific memory B cells before vaccination (T0). **(B)** Dot chart depicts the frequency of spike-specific memory B cells in the two groups before (T0) and 10 days after vaccination (T1). Concentration of anti-TrimericS IgG (BAU/mL) and neutralization titers, reported as IC50 against the Wuhan viral strain are shown in **(C, D)**, respectively. **(E)** Graph indicates the inhibitory activity against the Omicron VOC in the two groups. **(F)** Bar charts represent the percentage of children in group 1 and group 2 with anti-TrimericS IgG (BAU/mL), neutralizing antibodies against the complete Wuhan spike and antibodies able to inhibit the binding of ACE-2 to the Omicron RBD. Dot charts show the number of T cells, expressed as numbers of spot-forming cells (SFC)/10^6^ PBMC, producing IFN gamma after stimulation with peptides encompassing the complete Wuhan spike protein (complete Wuhan spike) **(G)**, the spike region mutated in the Omicron VOC **(H)** and its unmutated counterpart **(I)**. **(J)** Bar charts represent the percentage of responders in group 1 and group 2 against the complete Wuhan spike protein, the spike region mutated in the Omicron VOC and its unmutated counterpart. Dashed lines indicate the cutoff value for each test. Bars indicate medians and dots with thick borders show values measured in children that had experienced COVID-19 before vaccination (n=3). Non-parametric Wilcoxon matched pair signed-rank test (continuous line) and Mann–Whitney t-test (dashed line) were used to evaluate statistical significance. Categorical variables were compared by Chi-Square exact test. Two-tailed P value significances are shown as * p<0.05, **p < 0.01, ***p < 0.001, ****p < 0.0001.

Fifteen (55.5%) of the children in our cohort had spike-specific memory B cells at T0 (**Figure 3** **A**). As only three of them had a documented history of infection, 50% (12/24) of the remaining children had probably experienced an infection that was never detected, although NPS was performed in the subjects who had contact with infected individuals or presented respiratory symptoms.

Anti-N antibodies are considered a reliable indicator of a previous SARS-CoV-2 infection. We found that anti-N antibodies were, however, present only in 73% of the children of group 1 (**Supplementary Figure 2A**). Anti-N antibodies were undetectable in 27% of group 1, including two children who had experienced COVID-19 more than one year before. The progressive decline of anti-N IgG was confirmed by their reduction at T1, about 30 days after the T0 measurement (**Table 1** and **Supplementary Figure 2B**). Thus, due to their continuous decline, anti-N antibodies may fail to identify children who had been infected a long time before the serological test.

We found that spike-specific memory B cells (**Figure 3B**) increased in both groups after vaccination, but the response was significantly greater in group 1 than in group 2 (p=0.0002).

Children of group 1 also had higher levels of anti-TrimericS IgG (**Figure 3** **C**; p=0.001) and neutralizing antibodies (**Figure 3** **D**; p=0.04) after the 2nd dose.

It has been demonstrated that repeated exposures to the SARS-CoV-2 spike protein, either because of infection before or after vaccination or administration of a booster vaccine dose, increase immunity against not only the Wuhan strain but also the Omicron VOC (36).

In order to have a measure of the potential neutralization ability of vaccine-induced antibodies against the Omicron VOC, we measured their ability to inhibit the binding of ACE-2 to the Omicron RBD. The inhibitory activity was increased by vaccination in all children, but group 1 had higher inhibitory antibody titers before (p=0.006) and after vaccination (p=0.01) compared to group 2 (**Figure 3E**). Thus, although after vaccination all children had high titers of anti-TrimericS and neutralizing antibodies against the Wuhan strain, antibodies with inhibitory activity against the Omicron VOC were present in 87% (13/15) of the children in group 1 and only in 42% of those of group 2 (5/12) (p=0.01; **Figure 3F**).

Spike-specific T cells directed against the Wuhan strain spike (complete Wuhan spike) were equally induced in group 1 and group 2 children (**Figure 3G**), and the response rate after vaccination was 100% in both groups (**Figure 3J**). We also measured the frequency of T cells able to recognize the region mutated in the Omicron spike and, as control, its unmutated counterpart (Wuhan reference). We found that, after vaccination, children of group 1 had significantly better T-cell responses than those of group 2 (Omicron spike p=0.04; Wuhan reference p=0.01) (**Figures 3H, I**). Most importantly, only 18% (2/11) of the children in group 2 had T cells able to recognize the Omicron spike, whereas, in group 1, 71.4% (10/14) of the children were responders (**Figure 3J**). The Wuhan reference of the Omicron spike was recognized by 80% of the children in group 1 and 40% of those in group 2 (**Figure 3J**).

Thus, in response to vaccination, children who had spike-specific memory B cells at T0 produced more antibodies, had more memory B cells and antigen-specific T cells, and were able to react against the Omicron VOC, probably as a result of highly effective hybrid immunity (37). For children completely naïve to the virus, two vaccine doses may be insufficient to obtain the same degree of immunity.

## Discussion

Since its first description in 2019, SARS-CoV-2, the causative agent of COVID-19, continues to accumulate mutations and generate variants. Although children present an asymptomatic or paucisymptomatic course, complications from COVID-19 may occur and pediatric infection represents a public health problem. The highly transmissible Omicron VOC is now dominant all over the world. Although several studies have shown that the Omicron wave is associated with milder illness and an overall lower hospitalization rate (38, 39), the number of pediatric patients affected by Omicron exceeds the total number of cases seen in previous waves (40), ultimately leading to an increase in the absolute number of pediatric patients with hospitalization and severe outcomes (2). Vaccination is the best strategy to reduce the severity of the disease and limit complications of COVID-19. It has been shown that BNT162b2 vaccination can consistently reduce Omicron-associated hospitalizations in children (28, 41) and prevent or reduce the associated complications, such as MIS-C (42), long COVID (43), and impairment of social and mental wellbeing (44).

After being recommended for adolescents, the BNT162b2 mRNA vaccine was approved and found to be safe, immunogenic, and efficacious in children 5 to 11 years old (11, 45), and, most recently, in children with an age between 4 months and 4 years (13). The immune response to pediatric vaccination has not yet been fully elucidated, and specifically, an understanding of the role of cellular immunity to infection and vaccination is missing at this age (46). Indeed, the registration studies have focused on neutralizing antibody levels for immunobridging (11). Although antibodies are a reliable measure of vaccine efficacy, memory B and T cells are important for long-term protection and are capable of responding to emerging VOC (15).

We studied the humoral and cellular immune response to the BNT162b2 vaccine encoding the spike protein of the Wuhan viral strain. All children responded to the vaccine with a significant increase of anti-TrimericS IgG, neutralizing antibodies, spike-specific memory B cells and antigen-specific T cells (**Figure 2**).

We have previously published that, in adults (16, 17), spike-specific memory B cells are undetectable before vaccination and develop after a complete cycle, because they are generated by the complex mechanism that modifies immunoglobulin genes and increases antibody affinity.

Unexpectedly, we detected circulating spike-specific memory B cells before administration of the first dose in 15 of 27 children (**Figure 3**). Only three of them had a documented history of COVID-19, suggesting that in the other 12 individuals, memory B cells may have been generated by undetected and asymptomatic SARS-CoV-2 infections. All infections known (3 cases) and undiagnosed (12 cases) occurred before the Omicron variant spread in Italy. This hypothesis was confirmed by the presence of anti-TrimericS IgG, neutralizing antibodies, and Spike-specific T cells in the samples collected at T0 (**Figure 3**). Anti-N IgG were detectable in the majority, but not all the children who had pre-existing immunity to SARS-CoV-2, probably because of the anti-N antibodies physiological decline, months after the infection (35) (**Supplementary Figures 2A, B**). In comparison to children without pre-existing antigen-specific memory B cells, those with established B and T cells immunity had a response to the vaccine that was not only significantly stronger but also broader, as demonstrated by the presence of antibodies with inhibitory activity against the Omicron VOC and T cells specific for the mutated regions of the Omicron Spike (**Figure 3**).

Our data demonstrate that the presence of spike-specific memory B cells identifies individuals who had a previous undiagnosed encounter with the virus. Based on the information obtained from the structured questionnaire that was administered to the parents to investigate whether the children had had positive NPS or experienced COVID-19 before, 80% (12/15) of the children with pre-existing immunity had experienced an asymptomatic and undiagnosed infection. This infection had, however, generated persistent memory B and T cells, responsible for the strong reaction to the vaccine, typical of hybrid immunity.

As a broader response, anti-Omicron neutralizing antibodies are induced after a booster dose in adults and adolescents (47, 48), our results suggest that a third vaccine dose may amplify the amount and breadth of vaccine-induced immunity also in children. A primary response to immunization is directed against the more immunogenic dominant epitopes (49). Repeated exposures to the spike protein, because of booster doses or natural infection, may redirect the response to other regions of the immunizing antigen and thus explain the increase of Omicron neutralizing antibodies (50) and of T cells recognizing the Spike regions mutated in the Omicron VOC (51) (**Figure 3**).

The CDC has approved the administration of a homologous booster dose for children aged 5–11 years and reviewed the data demonstrating the safety of the procedure (52). Most recently, bivalent COVID-19 booster doses have been authorized in the USA also for children ages 5 and above, with the aim of broadening and strengthening the protection against the currently dominant VOC. Furthermore, a recent study indicates that protection against Omicron infection generated by two mRNA vaccine doses or by infection rapidly declines, suggesting that children may benefit from a booster dose of vaccine (53). In Italy, the third dose, either with the homologous or bivalent vaccine, has not yet been approved for children of this age.

The SARS-CoV-2 pandemic has been caused by the lack of pre-existing immunity to a virus never encountered before by humans. Vaccines have reduced the number of severe cases and deaths thanks to the generation of immune memory. Although so far, all vaccinated people are all immunized against the Wuhan Spike protein, vaccine-induced memory B and T cells are also able to react to the Omicron variant.

The major strength of our study is the comprehensive evaluation of the immune response to COVID-19 vaccination in 5 to 11 years old children. Until now, all published studies have measured serum antibodies. Here, we also demonstrate that B and T cell memory is elicited and that repeated antigen encounters lead to a broader response directed against the Wuhan virus and also the Omicron VOC. We also show that spike-specific memory B cells are a reliable indicator of a previous infection.

Our work has major limitations. We analyzed a limited number of subjects due to the difficulty of recruiting children in a study that requires repeated blood sample collections. We did not evaluate the neutralization activity of vaccine-induced antibodies against Omicron with the classic neutralization test, because the system was not available in our laboratories. We, however, measured the ability of vaccine-induced antibodies to block the binding of ACE-2 to the Omicron RBD, thus measuring specificity and activity against Omicron. Evaluation of memory B cells specific for Omicron was impossible due to the small amount of blood available for our study.

In conclusion, we provide the demonstration that the BNT162b2 vaccine is immunogenic in children and repeated antigen exposures may increase the ability to react toward the emerging VOC. The results of this study offer new insights into the humoral and cellular response to SARS-CoV-2 mRNA vaccines in children, useful to support public health decisions on the definition of future vaccination strategies in the pediatric age. Improving knowledge on immune response following vaccination may also help overcome vaccine hesitancy, considering that children, as adults, have the right to be protected from severe disease and COVID-19 complications and sequelae (44).

## Data availability statement

The raw data supporting the conclusions of this article will be made available by the authors, without undue reservation.

## Ethics statement 

The studies involving human participants were reviewed and approved by Ethical Review Committee of Sapienza, University of Rome, Italy (Prot. 0254/2022). Written informed consent to participate in this study was provided by the participants’ legal guardian/next of kin.

## Author contributions

BLC, EPM, VB, ChrA, CA, GF, IS, PS, SF, and MC acquired the data. BLC, EPM and RC were responsible for the analysis of the data. BLC, EPM and RC drafted the article. AMZ, CA, MGC, LP, ATP, PS, AS, FM, IS, FL critically revised the manuscript for important intellectual content. All authors approved the final version to be published and agree to be held accountable for all aspects of the work. BLC, EPM and RC had full access to, and verified, all the data in the study, and had final responsibility for the decision to submit for publication. RC, CA and FL made substantial contributions to the acquisition of funding. All authors contributed to the article and approved the submitted version.
