# Supplementary material for: The BNT162b2 vaccine induces humoral and cellular immune memory to SARS-CoV-2 Wuhan strain and the Omicron variant in children 5 to 11 years of age
Source: Front Immunol. 2022 Dec 15;13:1094727. doi: 10.3389/fimmu.2022.1094727 (PMC9797965; doi:10.3389/fimmu.2022.1094727)
Supplement: Supplementary file 1 [file DataSheet_1.pdf]

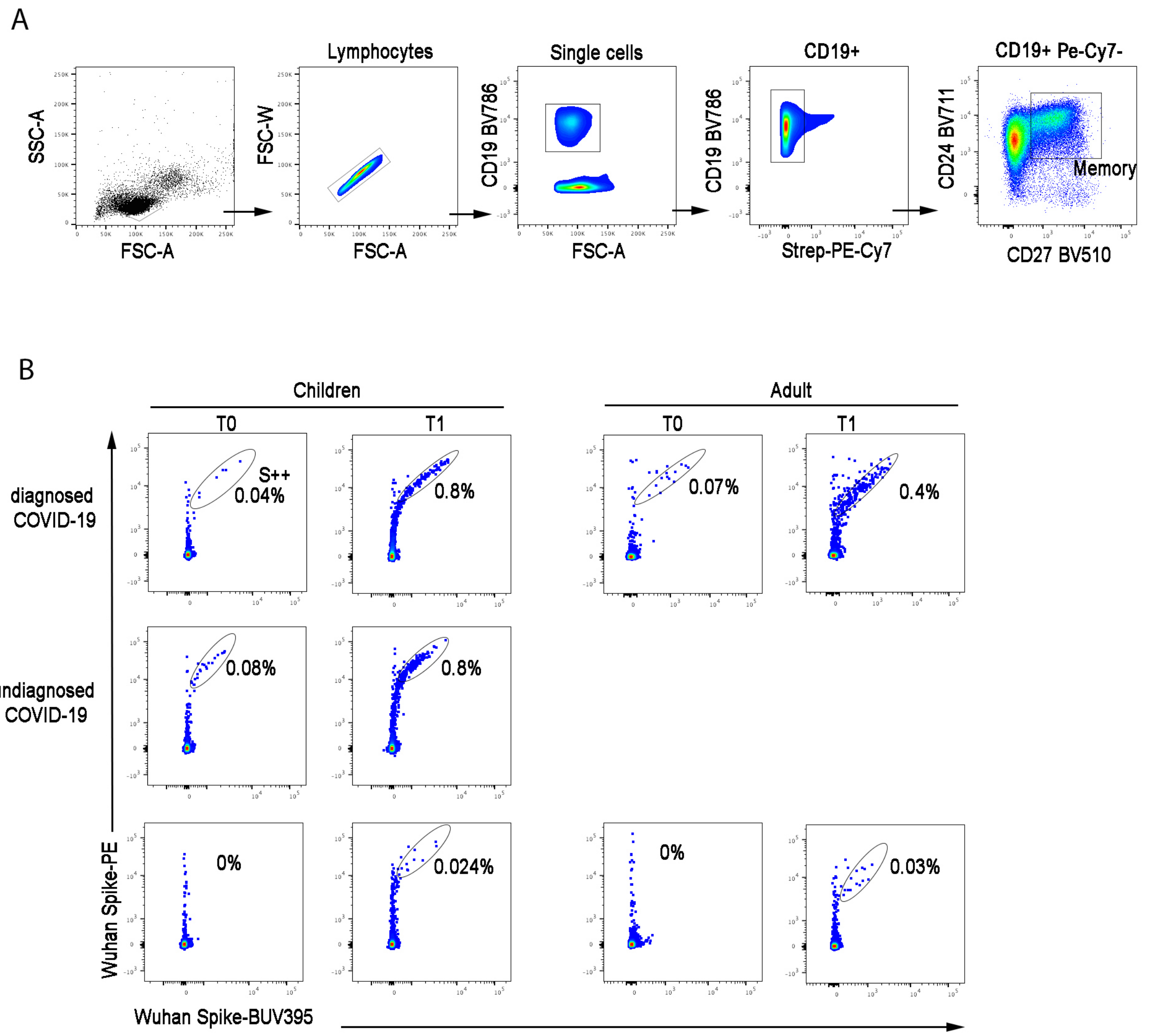

Supplementary Figure 1

Supplementary Figure 1: (A) FACS plots depict the gating strategy for the identification of total memory B cells (CD19+CD24+CD27+), and (B) inside memory B cells the spike specific memory B cells in children and adults with or without previous SARS-CoV-2 infection before (T0) and after vaccination (T1).

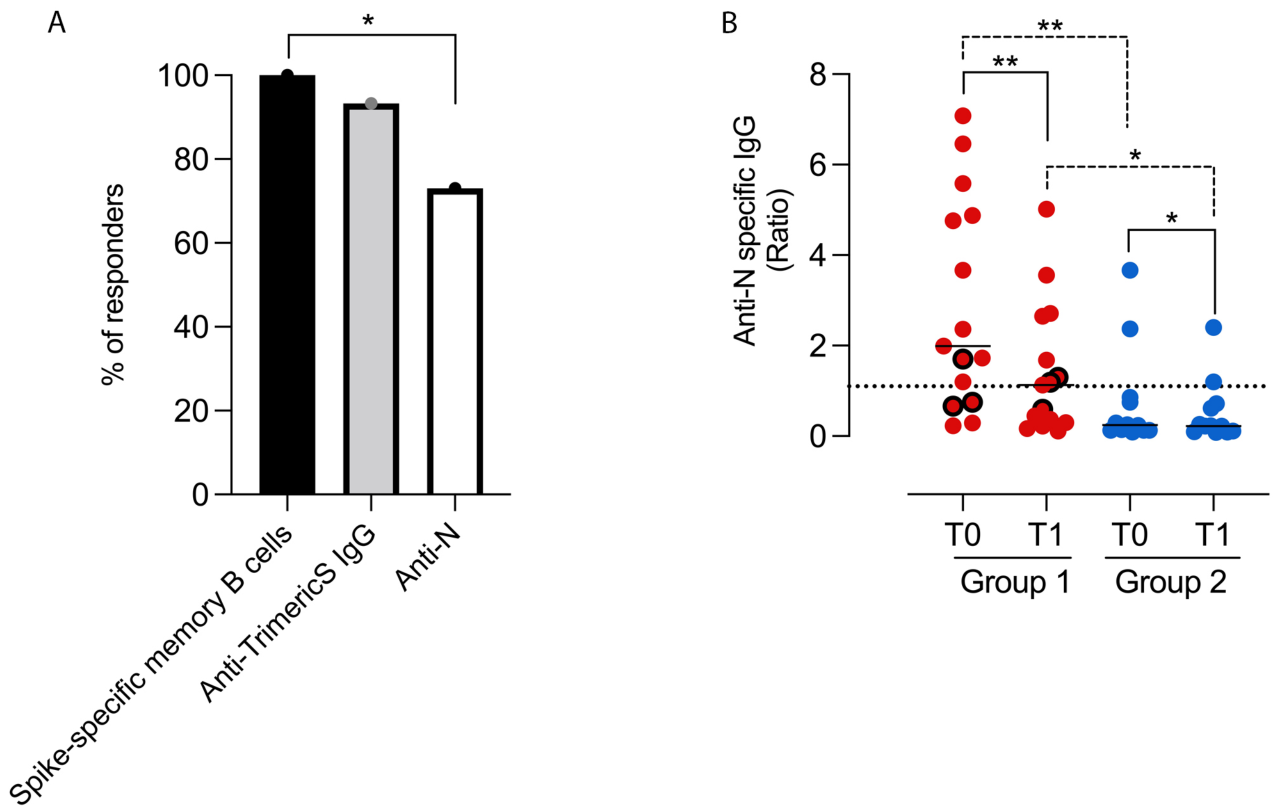

Supplementary Figure 2: (A) Percentage of children in group 1 who had Spike-specific memory B cells, anti-TrimericS IgG and anti-N antibodies at T0. (B) Dot chart depicts the concentration of anti-N IgG in children with (group 1) or without (group 2) spike-specific memory B cells before (T0) and 10 days after a complete vaccination cycle. Dashed lines indicate the cutoff value for each test. Bars indicate median and dots with thick borders show values measured in children with documented COVID-19 before vaccination (n=3). Non-parametric Wilcoxon matched pair signed-rank test (continuous line) and Mann–Whitney t-test (dashed line) were used to evaluate statistical significance. Categorical variables were compared by Chi-Square exact test. Two-tailed P value significances are shown as * $p<0.05$, ** $p < 0.01$
